# Clinical value of delayed ^18^F-FDG PET/CT for predicting nipple-areolar complex involvement in breast cancer: A comparison with clinical symptoms and breast MRI

**DOI:** 10.1371/journal.pone.0203649

**Published:** 2018-09-12

**Authors:** Jang Yoo, Bom Sahn Kim, Jin Chung, Hai-Jeon Yoon

**Affiliations:** 1 Department of Nuclear Medicine, Ewha Womans University School of Medicine, Seoul, South Korea; 2 Sungkyunkwan University School of Medicine, Seoul, South Korea; 3 Department of Nuclear Medicine, Veterans Health Service Medical Center, Seoul, South Korea; 4 Department of Radiology, Ewha Womans University, School of Medicine, Seoul, South Korea; Syddansk Universitet, DENMARK

## Abstract

**Objective:**

We aimed to evaluate the predictive value of delayed ^18^F-FDG PET/CT for identifying malignancies involved in the nipple-areolar complex (NAC) in comparison with clinical symptoms and breast MRI.

**Methods:**

We enrolled 90 patients who underwent preoperative delayed ^18^F-FDG PET/CT and MRI between October 2015 and May 2017. We calculated the NAC-Standardized uptake value ratio (SUVR) using the following formula: maximum SUV (SUV_max_) of the NAC in the malignant breast /SUV_max_ of the NAC in the contralateral normal breast on early (NAC-SUVR_early_) and delayed (NAC-SUVR_delay_) phase images. MRI was used to measure the distance between the tumor and NAC and to analyze NAC enhancement patterns. Univariate and multivariate analyses were performed to identify significant predictive factors for NAC involvement.

**Results:**

Seventeen patients were confirmed to have pathologic NAC involvement. NAC symptoms (p = 0.009), tumor multiplicity (p = 0.006), NAC-SUVR_delay_ (> 1.23, p = 0.007), and MRI-based tumor-to-NAC distance (≤ 22.0 mm, p = 0.003) were independent predictive factors for NAC involvement. Ten of 76 patients with no clinical NAC symptoms had NAC malignancy. Tumor multiplicity (p = 0.009), tumor-to-NAC distance (≤ 20.0 mm, p = 0.014)), and NAC-SUVR_delay_ (> 1.23, p = 0.018) had independent predictive value for NAC involvement.

**Conclusions:**

Delayed ^18^F-FDG PET/CT is a useful modality for predicting NAC involvement in breast cancer patients whether or not NAC symptoms are present.

## Introduction

Although surgical techniques for breast cancer have developed over the past few years to include less invasive and more cosmetically acceptable approaches, there remains continued interest in improving cosmetic results and oncologic safety [1]. Breast-conserving surgery (BCS) is widely performed, especially in patients with small breast cancer tumors with absence of skin, nipple, or chest involvement [2, 3]. With adjuvant radiotherapy, BCS provides equivalent results in patient survival and local recurrence compared to mastectomy. Although BCS is an attractive alternative surgical procedure to mastectomy, not all patients are eligible. For patients who require mastectomy, breast reconstruction can be performed immediately or delayed until after mastectomy. Conservative mastectomies were introduced in the early 1990s and, combined with breast reconstruction, yield superior cosmetic outcomes for better reconstructed breast contour and preservation of the inframammary fold [4–6]. For breast reconstruction after mastectomy, predicting tumor cell involvement in the nipple-areolar complex (NAC) can be a major issue affecting patient emotional and cosmetic satisfaction and oncologic safety [7, 8]. Recently, there has been significant evolution in nipple-areolar sparing mastectomy (NSM) as a conservative surgical approach, with equivalent local control of disease [9]. NAC involvement is an added challenge in invasive breast cancer cases, particularly in selecting patients who are appropriately indicated for NSM for cosmetic and aesthetic reasons. Several studies have assessed NAC involvement according to clinical and pathological characteristics [10–14]. To the best of our knowledge, however, there have been no investigations into the efficacy of ^18^F-fluorodeoxyglucose (FDG) positron emission tomography/computed tomography (PET/CT) for detecting NAC involvement.

Standard PET/CT for initial staging work-up is generally performed with the patient in a supine position. However, the prone position PET/CT could provide better visualization of breast lesions than the supine position [15, 16]. Several studies have demonstrated that delayed ^18^F-FDG PET/CT could be useful for improving diagnostic accuracy for breast cancer since radiopharmaceutical uptake in cancer cells could remain or even become enhanced over time [17, 18].

Since nipple symptoms (e.g., nipple erosion, discharge, retraction) are highly suspicious for NAC invasion, there is a major concern that the possibility of NAC involvement might be greater in patients who suffer from nipple symptoms than in those who are symptom-free. We assumed that it would be more challenging to identify NAC involvement in nipple symptom-free patients than in those with nipple symptoms. In patients with invasive breast cancer, therefore, we aimed to demonstrate the predictive value of preoperative prone position delayed ^18^F-FDG PET/CT and magnetic resonance imaging (MRI) parameters for determining NAC involvement compared with clinicopathologic factors according to the presence and/or absence of nipple symptoms.

## Materials and methods

### Patients and clinicopathologic analysis

Between October 2015 and May 2017, 129 female patients with suspected centrally located breast cancer underwent preoperative prone position dual time point (DTP) PET/CT and dynamic contrast-enhanced breast MRI. A centrally located tumor was defined as a cylinder of breast tissue beneath the NAC from the dermis to the pectoral fascia, based on a previous study [19]. We excluded 21 patients who had received neoadjuvant chemotherapy and 12 patients who did not undergo surgery. Three patients were excluded because they received surgical excisional biopsy before PET/CT, and three patients were excluded for having bilateral breast cancer. Ultimately, 90 patients were enrolled in this study.

We recorded patient ages and clinical NAC symptoms, including nipple pain, nipple discharge, retraction, and erosion, based on our institutional medical database. Pathologic variables of tumor size, axillary lymph node status (ANS), lymphovascular invasion (LVI), combined *in situ* components, and tumor multiplicity were reviewed based on final pathologic reports. Tumor size was defined as the maximum diameter of the surgical specimen. In cases of multifocal/multicentric breast cancer, the diameter of the largest lesion was selected. For examining ANS, either sentinel lymph node biopsy (SLNB) or axillary lymph node dissection (ALND) was performed according to the patient’s surgical plan and clinical nodal stages. When SLNBs were identified as metastasis, ALND was conducted, including axillary levels I and II. If nodal metastasis was clinically suspected or there were cases for which SLNB was not necessary, surgical removal was performed for determining the pathologic ANS.

Pathologic NAC involvement was determined by the presence of invasive or *in situ* tumor cells in the NAC or nipple ducts or Paget’s disease. To evaluate suspicious NAC involvement from a physical examination or imaging work-up, surgical biopsy of subareolar tissue or resection margins of the nipple were done. The subareolar tissue is a thin layer specimen located below the NAC. Intraoperative frozen sections of the subareolar tissue or resection margins were sent to a pathologist. Corresponding specimens were stained with the hematoxylin and eosin method. When specimens showed no evidence of invasive or carcinoma *in situ*, we considered this as negative pathologic finding. Otherwise, in operative specimens involving the NAC by mastectomy, the NAC was evaluated using a single plane dissection in 2–3 mm intervals perpendicular to the skin surface and was submitted perpendicularly for microscopic examination. The identification of cancer cells in these sections was regarded as pathologic NAC involvement. All surgical treatments and pathologic examinations were performed in our hospital, and we considered the final surgical pathology as the standard reference.

This prospective study was approved by the institutional review board of Ewha Womans University Hospital, and written informed consent was obtained from all participants.

### Dual time point ^18^F-FDG PET/CT and image analysis

All patients were asked to fast for at least six hours, and blood glucose level was required to be less than 140 mg/dL before intravenous ^18^F-FDG administration of 5.18 MBq/kg. Patients were also asked to rest for one hour after radioisotope administration, and non-contrast CT was obtained for attenuation correction, followed by a PET scan using a Siemens Biograph mCT with 128-slice CT (Siemens Medical Solutions, Knoxville, TN, USA). Torso images were acquired from the skull base to the proximal thigh with the patient in the supine position. Additional breast images were performed with the patient in the prone position using a mock-up coil for hanging both breasts (Breast Protector^™^, Body Support Systems, Inc., USA). Two hours after radioisotope injection, delayed phase images were acquired to obtain corresponding images of the breast while the patient remained in the prone position. DTP PET/CT images were interpreted by two nuclear physicians (J.Y and B.S.K with 8 and 14 years of experience, respectively) using a dedicated workstation (syngo.via, Siemens Medical Solutions); the physicians were blinded to all clinicopathologic information and radiologic findings. Qualitative interpretations were performed for the presence of enhanced FDG uptake, and we considered a PET/CT finding to be positive if there was asymmetric FDG uptake of the nipple that extended into the subareolar and/or periareolar area of the malignant breast.

For quantitative analysis, the maximum standardized uptake value (SUV_max_) was corrected for each individual patient’s body weight and was measured by delineating a three-dimensional volume of interest (VOI) over the NAC. The most representative image was selected, and the VOIs were drawn carefully to not include contiguous lesion areas. SUV_max_ values for the NAC in the malignant breast and the contralateral normal breast were obtained, and we then calculated the NAC-SUV_ratio_ with the following formula: NAC-SUV_ratio_ = SUV_max_ of the NAC in the malignant breast/SUV_max_ of the NAC in the contralateral normal breast. The NAC-SUV_ratio_ was calculated for early (NAC-SUVR_early_) and delayed (NAC-SUVR_delay_) phase images.

### MRI protocol and image analysis

Preoperative breast MRI was performed using the 3.0 Tesla (T) Achieva system (Philips Medical System, Best, The Netherlands). A dedicated breast coil (SENSE BREAST 7 Coil) was used with the patient in a prone position. An axial, fat-suppressed, fast-echo T2-weighted image was acquired in advance (TR/TE = 5521/70; flip angle = 90°; field of view = 320 mm; matrix = 332/261; slice thickness = 3 mm with no gap; bandwidth = 289 Hz; time acquisition = 4 min 23 s). Dynamic MRI using an enhanced T1-weighted high resolution isotropic volume excitation sequence was obtained after continuous intravenous injection of a gadolinium bolus (0.1 mmol/kg of body weight of Gd-DTPA; Gadovist, Bayer Schering Pharma AG, Berlin, Germany) at a rate of 2 ml/s, followed by a 25 ml saline flush using an automatic injector. Six phases of dynamic enhanced images were processed at 55.4 s (axial), 110.8 s (axial), 146 s (sagittal), 221.6 (axial), 292 s (sagittal), and 438 s (axial), respectively. The dynamic axial and sagittal MR variables were as follows: TR/TE = 4.42/2.17; flip angle = 12°; field of view = 320 mm; matrix = 320/320; receiver bandwidth = 621.4 Hz/pixel; slice thickness = 1 mm and 4.37/2.15; 12°; 25 cm; 250/250; 704.5 Hz/pixel; 1 mm.

The distance between the tumor and the NAC was measured from the center of the NAC to a representative tumor on both axial and sagittal MRI images; we defined this as the minimum distance between the NAC and the nearest tumor margin. The positive or negative MR finding for NAC involvement was determined according to linear enhancement of the NAC directly from the primary tumor, unilateral NAC enhancement, and asymmetric thickness of the NAC, in accordance with previous studies [20, 21]. Preoperative breast MRIs were interpreted by a radiology specialist (J.C) with 16 years of experience with breast MRI, and consensus was achieved in all patients.

### Statistical analysis

Statistical analysis was performed using the Medcalc software package (Version 9.5, Medcalc software). All continuous variables were recorded as mean value ± standard deviation (SD) or median value with interquartile range (IQR), depending on the data distribution. The Kolmogorov-Smirnov test was used to determine if the data were normally distributed. If a normal distribution was suspected, we used an independent t-test to compare continuous variables. If the variables did not appear to be normally distributed, we used the Mann-Whitney test. We also used Pearson’s chi-square test to compare categorical variables regarding NAC involvement.

To estimate the optimal cutoff values for continuous variables to predict NAC involvement, we performed receiver operating characteristic (ROC) curve analysis using the Youden index. Multivariate analysis using logistic regression models with a stepwise selection process was performed to identify independent predictors of NAC involvement. A p-value less than 0.05 was considered statistically significant.

## Results

### Patient characteristics

Ninety patients (mean age ± SD = 53.4 ± 12.7) were included in this study. The average interval time between PET/CT and MRI was 2.1 days. BCS was performed on 47 patients, modified radical mastectomy was performed on 30, and the remaining 13 patients underwent skin-sparing mastectomy. Seventy-seven patients had invasive ductal carcinoma, 5 had invasive lobular carcinoma, 3 had invasive mucinous carcinoma, 3 had invasive medullary carcinoma, 1 had invasive papillary carcinoma, and 1 had a malignant phyllodes tumor. Five patients had N3 stage, six had N2, and 29 had N1 based on the final pathologic results. Fifty patients had no evidence of axillary lymph node metastasis through SLNB or ALND. Twenty-seven of the 90 patients were evaluated for NAC involvement through the operative specimens involving NAC. The remaining 63 underwent surgical biopsy of subareolar tissue or resection margins of the nipple. Finally, seventeen patients (18.9%) were confirmed to have NAC involvement based on the pathology results.

Table 1 presents the results from univariate analysis of clinicopathologic factors according to NAC involvement. There were 14 patients who complained of NAC symptoms, comprising discharge (n = 1), retraction (n = 8), erosion (n = 3), and pain (n = 2). The presence of NAC symptoms had significant predictive value for NAC involvement (p = 0.004). Tumor multiplicity also revealed predictive significance for NAC invasion (p = 0.037).

**Table 1:** Univariate analysis of clinicopathologic factors according to NAC involvement.

Factor		Pathologic NAC involvement (n = 17)	No pathologic NAC involvement (n = 73)	P-value
Age (year)		49.5 ± 9.9	54.3 ± 13.3	0.144
NAC symptoms	Presence	7	7	0.004*
	Absence	10	66	
Pathologic tumor size (cm)	Median (IQR)	2.35 (1.35–3.90)	2.10 (1.28–3.00)	0.307
ANS	Positive	9	31	0.609
	Negative	8	42	
LVI	Positive	9	20	0.082
	Negative	8	53	
Combined *in situ* component	Positive	14	61	0.810
	Negative	3	12	
Tumor multiplicity	Yes	7	11	0.037*
	No	10	62	

NAC, nipple-areolar complex; ANS, axillary lymph node status; LVI, lymphovascular invasion; IQR, interquartile range;

*, p <0.05

Pathologic tumor size was not significantly different between patients with pathologic NAC involvement and those without (p = 0.307). LVI was more common in the NAC-involved group; however, the difference did not reach statistical significance (p = 0.082). There were also no significant differences with respect to patient age (p = 0.144), ANS (p = 0.609) or combined *in situ* components (p = 0.810).

### Prediction of NAC involvement by DTP PET/CT and MRI characteristics

The presence of either asymmetric FDG uptake in the nipple or extension to NAC based on visual analysis was not predictive for pathologic NAC involvement (p = 0.167; Table 2). In contrast, our quantitative analysis using SUV_max_ found that both early (p = 0.005) and delayed (p < 0.001) phase NAC-SUV_ratio_ were significantly different between the NAC-involved and non-involved groups. After ROC curve analysis, the optimal cutoff values for NAC-SUVR_early_ and NAC-SUVR_delay_ were 1.32 (area under curve (AUC), 0.721; 95% confidence interval (CI), 0.597–0.796) and 1.23 (AUC, 0.810; 95% CI, 0.700–0.876), respectively.

**Table 2:** Correlations between NAC involvement and DTP PET/CT and MRI characteristics.

Modality	Factor		Pathologic NAC involvement (n = 17)	No pathologic NAC involvement (n = 73)	p-value
PET/CT	Qualitative analysis	Positive	9	23	0.167
		Negative	8	50	
	NAC-SUVR_early_	Median (IQR)	1.33 (1.12–1.59)	1.06 (0.96–1.27)	0.005*
	NAC-SUVR_delay_	Median (IQR)	1.42 (1.15–2.28)	1.04 (0.95–1.21)	<0.001*
MRI	Tumor-to-NAC distance (mm)	Mean ± SD	13.7 ± 8.7	29.1 ± 16.7	<0.001*
	MR finding	Positive	14	33	0.013*
		Negative	3	40	

NAC, nipple-areolar complex; DTP PET/CT, dual time point positron emission tomography/computed tomography; MRI, magnetic resonance imaging; NAC-SUVR_early_, ratio of early phase maximal standardized uptake value (SUV_max_) of the NAC in the breast with malignancy to that of the NAC in the contralateral normal breast; NAC-SUVR_delay_, ratio of delayed phase SUV_max_ of the NAC in the malignant breast to that of the NAC in the contralateral normal breast; IQR, interquartile range; SD, standard deviation;

*, p <0.05

Positive MRI finding (p = 0.013) including either linear enhancement to NAC, unilateral NAC enhancement and/or asymmetric thickening of NAC was significant predictor of NAC involvement. As shown in Table 2, MRI-based distance from the tumor to the NAC was also significantly different between the two groups (p < 0.001). The optimal cutoff value for the distance from the tumor to the NAC for predicting NAC involvement was ≤ 22.0 mm (AUC, 0.795; 95% CI, 0.687–0.867). There were weak negative correlations between NAC-SUVR_early_ and tumor-to-NAC distance and between NAC-SUVR_delay_ and tumor-to-NAC distance (rho = -0.250, p = 0.018 and rho = -0.259, p = 0.014, respectively).

**Fig 1:**
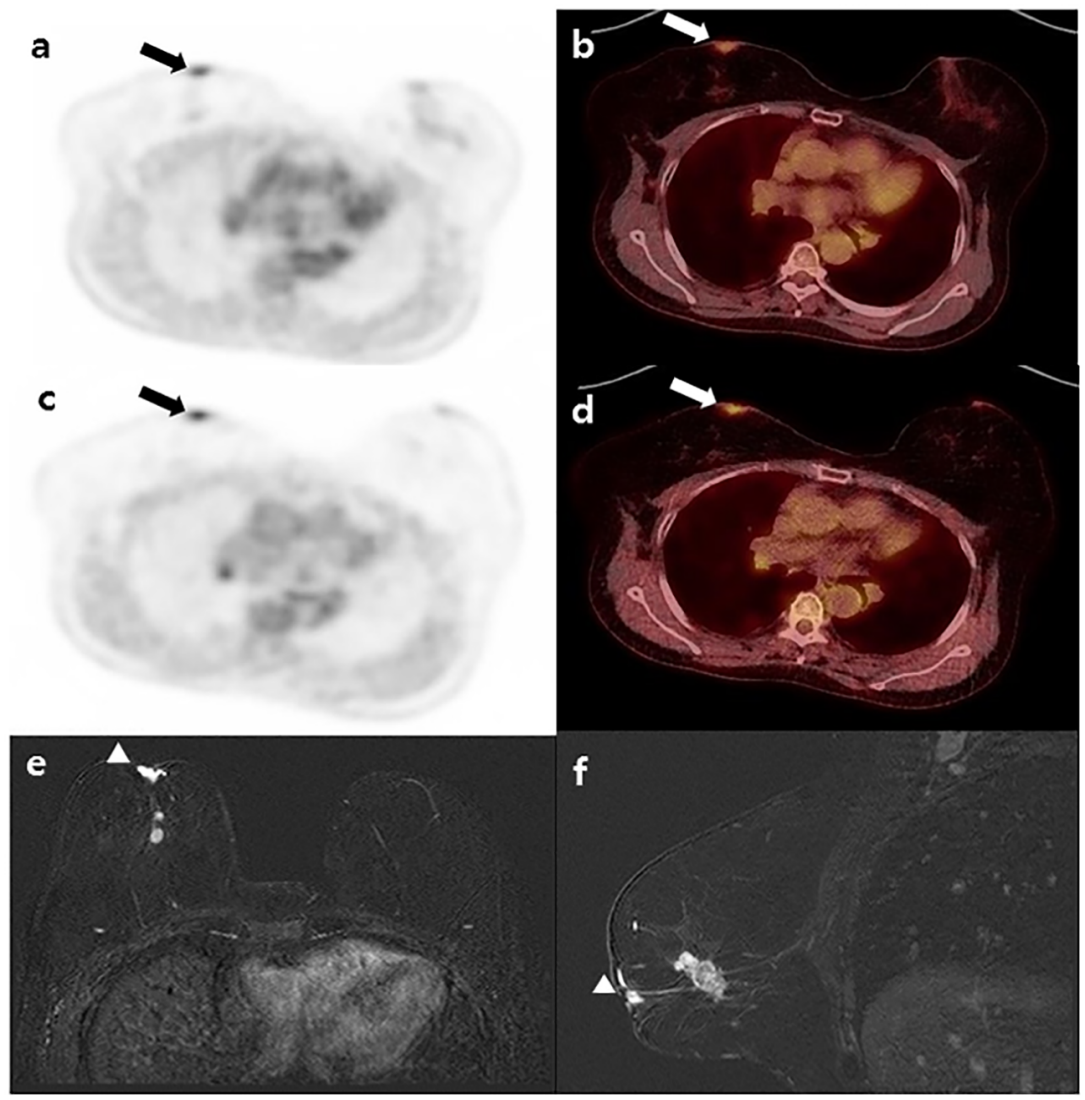
A 51-year-old patient with invasive ductal carcinoma (IDC) in the right breast: (a, b) Early phase ^18^F-FDG PET and fusion PET/CT; (c, d) Delayed phase PET and fusion PET/CT depict focal FDG uptake in the NAC of the right breast (arrow; ROI of NAC showing green color), NAC-SUVR_early_ = 2.35; NAC-SUVR_delay_ = 2.95; (e) Contrast-enhanced MRI with axial view and (f) MRI with sagittal view; MRI shows unilateral NAC enhancement and asymmetric thickening of the NAC (arrowhead). This patient underwent mastectomy, and NAC involvement was pathologically confirmed.

**Fig 2:**
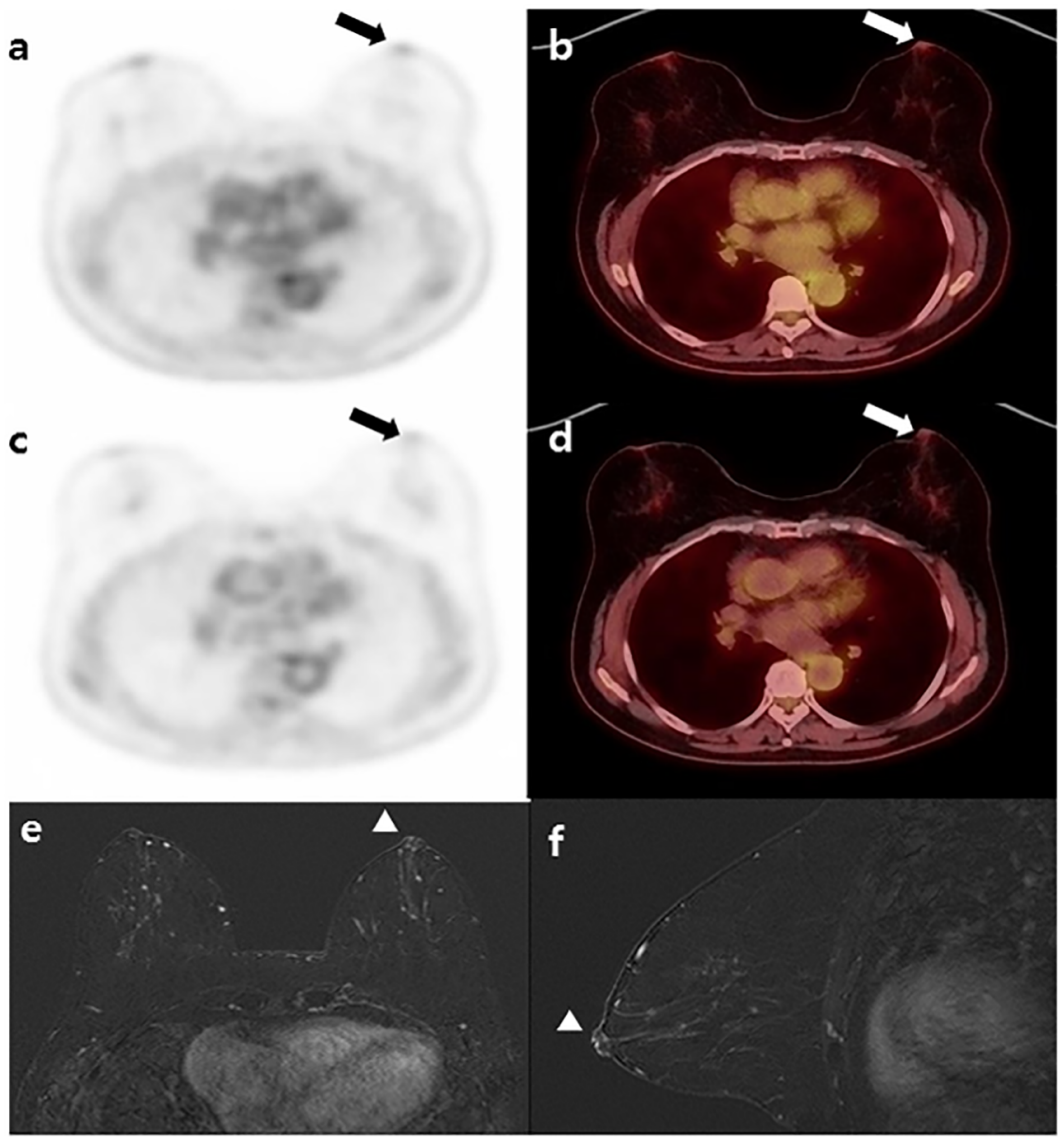
A 48-year-old patient with IDC in the left breast: (a, b) Early phase ^18^F-FDG PET and fusion PET/CT; (c,d) Delayed phase PET and fusion PET/CT show no significant FDG uptake in the NAC of the left breast (arrow), NAC-SUVR_early_ = 0.95; NAC-SUVR_delay_ = 0.92; (e) Contrast-enhanced MRI with axial view and (f) MRI with sagittal view; MRI reveals no definite evidence of abnormal contrast enhancement of the NAC in the left breast (arrowhead). This patient underwent breast conserving surgery, and pathologic NAC non-involvement was confirmed.

Our multivariate analysis demonstrated that NAC symptoms, tumor multiplicity, DTP PET/CT parameter of NAC-SUVR_delay_, and MRI-based distance from the tumor to the NAC had independent predictive value for NAC involvement (Table 3). According to these results, patients with NAC symptoms, tumor multiplicity, NAC-SUVR_delay_ higher than 1.23 or a central tumor located equal to or less than MRI-based 22.0 mm from the NAC had a risk of NAC involvement that was 22.08, 32.35, 20.30, and 93.42 times higher than their opposite groups, respectively. Representative cases of pathologic NAC involvement are illustrated in Figs 1 and 2.

**Table 3:** Multivariate analysis for predicting NAC involvement.

Variable	Cutoff value	OR	95% CI	p-value
NAC symptoms		22.08	2.148–226.983	0.009*
Tumor multiplicity		32.35	2.707–386.649	0.006*
NAC-SUVR_delay_	> 1.23	20.30	2.317–177.886	0.007*
Tumor-to-NAC distance (mm)	≤ 22.0	93.42	4.647–1877.901	0.003*

NAC, nipple-areolar complex; NAC-SUVR_delay_, ratio of delayed phase SUV_max_ of the affected NAC to that of the unaffected NAC; OR, odds ratio; CI, confidence interval;

*, p <0.05

### Clinical application of imaging parameters for NAC symptom-free patients

There were 76 patients who did not complain of nipple symptoms. Among them, 10 patients (13.2%) had confirmed malignant NAC involvement from final pathologic reports. Table 4 presents predictive variables for NAC involvement in NAC symptom-free patients. Tumor multiplicity, both DTP PET/CT parameters, tumor-to-NAC distance had significant predictive value in univariate analysis.

**Table 4:** Univariate analysis for predicting NAC involvement in nipple symptom-free patients.

Factor		Pathologic NAC involvement (n = 10)	No pathologic NAC involvement (n = 66)	P-value
Age (year)		45.5 ± 10.2	51.3 ± 12.5	0.463
Pathologic tumor size (cm)	Median (IQR)	2.45 (0.70–3.30)	2.10 (1.20–3.00)	0.516
ANS	Positive	5	28	0.914
	Negative	5	38	
LVI	Positive	4	18	0.651
	Negative	6	48	
Combined *in situ* component	Positive	9	57	0.853
	Negative	1	9	
Tumor multiplicity	Yes	6	9	0.003*
	No	4	57	
PET/CT				
Qualitative analysis	Positive	5	21	0.440
	Negative	5	45	
NAC-SUVR_early_	Median (IQR)	1.36 (1.12–1.57)	1.06 (0.96–1.27)	0.018*
NAC-SUVR_delay_	Median (IQR)	1.37 (1.15–2.38)	1.06 (0.95–1.22)	0.003*
MRI				
Tumor-to-NAC distance (mm)	Mean ± SD	15.5 ± 10.0	30.1 ± 17.0	0.010*
MR finding	Positive	8	28	0.060
	Negative	2	38	

NAC, nipple-areolar complex; ANS, axillary lymph node status; LVI, lymphovascular invasion; PET/CT, positron emission tomography/computed tomography; NAC-SUVR_early_, ratio of early phase maximal standardized uptake value (SUV_max_) of the NAC in the breast with malignancy to that of the NAC in the contralateral normal breast; NAC-SUVR_delay_, ratio of delayed phase SUV_max_ of the NAC in the malignant breast to that of the NAC in the contralateral normal breast; MRI, magnetic resonance imaging; IQR, interquartile range; SD, standard deviation;

*, p <0.05

After multivariate analysis, tumor multiplicity (p = 0.009), tumor-to-NAC distance (p = 0.014), and NAC-SUVR_delay_ (p = 0.018) were independently predictive (Table 5). Although the DTP PET/CT parameters of NAC-SUVR_early_ (p = 0.018) had predictive significance for NAC involvement in univariate analysis, neither maintained significance after logistic regression analysis. Positive MR finding could not show predictive significance in univariate analysis (p = 0.060).

**Table 5:** Multivariate analysis for predicting NAC involvement in nipple symptom-free patients.

Variable	Cutoff value	OR	95% CI	p-value
Tumor multiplicity		22.97	2.205–239.211	0.009*
Tumor-to-NAC distance (mm)	≤ 20.0	37.88	2.083–688.586	0.014*
NAC-SUVR_delay_	>1.23	15.45	1.598–149.297	0.018*

NAC, nipple-areolar complex; NAC-SUVR_delay_, ratio of delayed phase SUV_max_ of the affected NAC to that of the unaffected NAC; OR, odds ratio; CI, confidence interval;

*, p <0.05

## Discussion

An important finding from our analyses is that quantitative parameters derived from DTP PET/CT, such as both early and delayed phase SUV_max_ ratios of pathologically involved NACs compared to the contralateral normal breast, can offer significant predictive value for determining NAC involvement. However, only the delayed phase parameter appeared to be an independent predictor of NAC involvement in patients with centrally located breast cancer lesions. It is presumed that FDG uptake in cancer cells with an affected NAC would increase and persist on delayed phase images, while the contralateral normal NAC would wash out over time. Several reports have investigated the factors contributing to increased FDG uptake in breast cancer cells on delayed images [22–24].

This study revealed that breast MRI characteristics such as contrast enhancement pattern and distance between the NAC and tumor were correlated with NAC involvement, which is consistent with previous research [11, 20, 25]. Our results are also consistent with results from a previous study that found that clinical symptoms relevant to the NAC and tumor multiplicity were significantly more common in cases with NAC involvement than in those without [10, 14].

In addition, we demonstrated that delayed PET/CT parameter could also be significantly predictive for NAC involvement in breast cancer patients. In the literature of clinically silent NAC involvement, malignancy invasion to the NAC is detected in the range of 8–58% of mastectomy specimens [26–28]. In our study, 13.2% of patients with a clinically normal nipple were determined to have malignancy invasion to the NAC. Based on our findings, tumor multiplicity, tumor-to-NAC distance, and delayed phase of NAC-SUV_ratio_ against the contralateral normal NAC have independently predictive value for NAC involvement in both patient groups with nipple symptom and symptom-free. MR finding according to contrast enhancement patterns was not significantly predictive in the univariate analysis in the symptom-free subgroup, possibly because a considerable number of cases with NAC symptoms were excluded. To overcome this limitation, further study is needed for a larger group of symptom-free patients.

Our findings depart from those of other studies, since we found that tumor size, ANS, and LVI, which were correlated with NAC involvement in previous reports [12, 20, 25], were not significantly correlated with NAC involvement. We think that these differences are probably due to our relatively small number of research subjects or due to differences in subject populations.

There are several limitations in this study. First, our method for obtaining images with the patient in a prone position for PET/CT is not generally performed in other medical institutions. Supine whole-body image acquisition is still the standard protocol for assessing regional lymph node status and distant metastasis as well as for evaluating primary tumor lesions. Another limiting point may be the methodological problem of quantification. Because two consecutive tests were conducted at regular time intervals, there should be confidence in the high reproducibility of imaging quantification. Since previous studies [29, 30] showed that the mean SUV (SUV_mean_) has higher reproducibility than SUV_max_, it may be necessary to conduct research using SUV_mean_ in the future. DTP PET/CT was performed to acquire images approximately 80 and 120 minutes after administration of radiopharmaceutical agents. The mean ± SD time interval between early and delayed phase prone images was 45 ± 11 minutes. This protocol can lead to less accurate findings in non-avid cancer cells that affect the NAC because these images need to be obtained after longer time delays. However, the consensus for imaging acquisition time periods has not yet been established clinically. Lastly, we considered the pathologic results as the gold standard, including nipple biopsy and frozen sections. These accuracies could depend on variable situations, such as the indication, the location of the lesion, the quality of the pathologic slides, and the expertise of physician, which may cause false-negative findings [31]. This limitation may cause underestimated pathologic NAC involvement in the current study.

Despite these limitations, to the best our knowledge, ours is the first study to evaluate the predictive significance of quantitative parameters for NAC involvement using delayed PET/CT with patients in the prone position and to compare these findings with MRI findings and clinicopathologic factors. In conclusion, quantitative analysis of delay phased PET/CT might aid surgeons in deciding whether nipple saving is possible, as evidenced by NAC involvement, preoperative physical examination, and MRI features. In fact, one patient who had a false negative result according to MRI findings was indicated to be a true positive for NAC involvement according to PET/CT parameters.

Herein, we conclude that quantitative parameters from delayed PET/CT when patients are in a prone position can be used to complement clinicopathologic and MRI factors that are associated with malignant NAC involvement. Regardless of the presence of nipple symptoms on physical examination, delayed PET/CT could be performed for evaluating malignant NAC involvement at the preoperative work-up.

## Supporting information

S1 Table MRI interpretations of study subjects (n = 90). (DOCX)
