# Supplementary material for: Clinical value of delayed 18F-FDG PET/CT for predicting nipple-areolar complex involvement in breast cancer: A comparison with clinical symptoms and breast MRI
Source: PLoS One. 2018 Sep 12;13(9):e0203649. doi: 10.1371/journal.pone.0203649 (PMC6135503; doi:10.1371/journal.pone.0203649)
Supplement: S1 Table — (DOCX) [file pone.0203649.s001.docx]

S1 Table. MRI interpretations of study subjects (n = 90)

| MRI interpretations | Linear enhancement to NAC | Unilateral NAC enhancement | Asymmetric thickening of NAC |
| --- | --- | --- | --- |
| 1 | No | No | No |
| 2 | No | No | No |
| 3 | No | No | No |
| 4 | No | Yes | Yes |
| 5 | Yes | No | Yes |
| 6 | Yes | Yes | Yes |
| 7 | Yes | No | No |
| 8 | Yes | No | No |
| 9 | Yes | Yes | Yes |
| 10 | Yes | No | No |
| 11 | Yes | No | No |
| 12 | Yes | No | No |
| 13 | Yes | Yes | Yes |
| 14 | Yes | Yes | Yes |
| 15 | Yes | Yes | Yes |
| 16 | Yes | Yes | Yes |
| 17 | Yes | Yes | Yes |
| 18 | No | No | No |
| 19 | No | No | No |
| 20 | No | No | No |
| 21 | No | No | No |
| 22 | No | No | No |
| 23 | No | No | No |
| 24 | No | No | No |
| 25 | No | No | No |
| 26 | No | No | No |
| 27 | No | No | No |
| 28 | No | No | No |
| 29 | No | No | No |
| 30 | No | No | No |
| 31 | No | No | No |
| 32 | No | No | No |
| 33 | No | No | No |
| 34 | No | No | No |
| 35 | No | No | No |
| 36 | No | No | No |
| 37 | No | No | No |
| 38 | No | No | No |
| 39 | No | No | No |
| 40 | No | No | No |
| 41 | No | No | No |
| 42 | No | No | No |
| 43 | No | No | No |
| 44 | No | No | No |
| 45 | No | No | No |
| 46 | No | No | No |
| 47 | No | No | No |
| 48 | No | No | No |
| 49 | No | No | No |
| 50 | No | No | No |
| 51 | No | No | No |
| 52 | No | No | No |
| 53 | No | No | No |
| 54 | No | No | No |
| 55 | No | No | No |
| 56 | No | No | No |
| 57 | No | No | No |
| 58 | No | No | Yes |
| 59 | Yes | No | No |
| 60 | Yes | No | No |
| 61 | Yes | No | No |
| 62 | Yes | No | No |
| 63 | No | No | Yes |
| 64 | No | No | Yes |
| 65 | No | Yes | No |
| 66 | No | No | Yes |
| 67 | No | Yes | No |
| 68 | No | No | Yes |
| 69 | No | No | Yes |
| 70 | No | No | Yes |
| 71 | No | No | Yes |
| 72 | No | Yes | No |
| 73 | Yes | No | No |
| 74 | Yes | No | No |
| 75 | Yes | No | Yes |
| 76 | Yes | No | No |
| 77 | Yes | No | Yes |
| 78 | Yes | No | No |
| 79 | Yes | No | No |
| 80 | Yes | No | No |
| 81 | Yes | No | No |
| 82 | Yes | No | No |
| 83 | Yes | No | No |
| 84 | Yes | No | No |
| 85 | Yes | No | No |
| 86 | Yes | Yes | No |
| 87 | Yes | No | Yes |
| 88 | Yes | No | No |
| 89 | Yes | No | No |
| 90 | Yes | No | No |

MRI, magnetic resonance imaging; NAC, nipple-areolar complex
